# Radiometal-Labeled Photoactivatable Pt(IV) Anticancer Complex for Theranostic Phototherapy

**DOI:** 10.1021/acs.inorgchem.3c02245

**Published:** 2023-08-29

**Authors:** Cinzia Imberti, Jamie Lok, James P. C. Coverdale, Oliver W. L. Carter, Millie E. Fry, Miles L. Postings, Jana Kim, George Firth, Philip J. Blower, Peter J. Sadler

**Affiliations:** † Department of Chemistry, University of Warwick, Coventry CV4 7AL, U.K.; ‡ School of Pharmacy, Institute of Clinical Sciences, University of Birmingham, Birmingham B15 2TT, U.K.; § School of Biomedical Engineering & Imaging Sciences, King’s College London, St Thomas’ Hospital, London SE1 7EH, U.K.

## Abstract

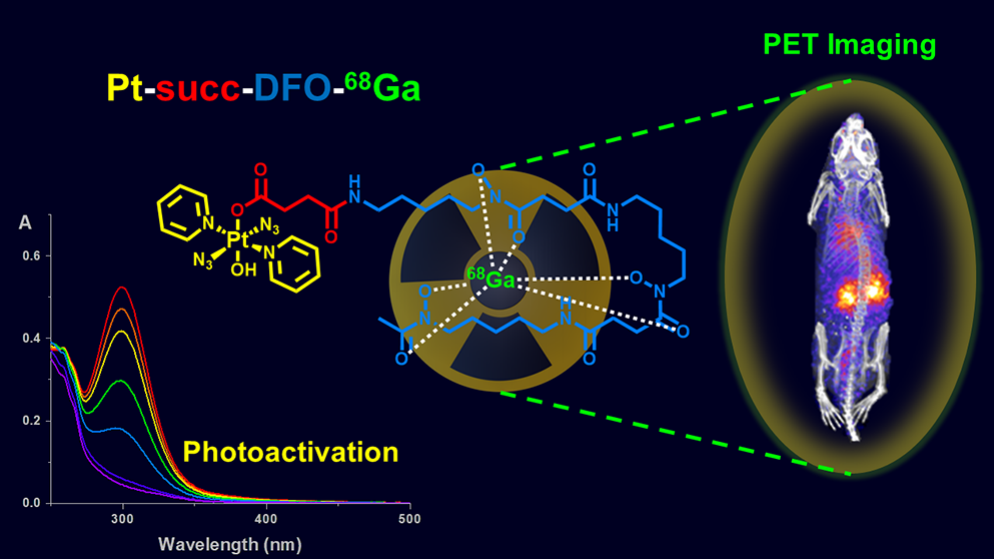

A novel photoactivatable Pt(IV) diazido anticancer agent, **Pt-succ-DFO**, bearing a pendant deferoxamine (DFO) siderophore for radiometal chelation, has been synthesized for the study of its *in vivo* behavior with radionuclide imaging. **Pt-succ-DFO** complexation of Fe(III) and Ga(III) ions yielded new heterobimetallic complexes that maintain the photoactivation properties and photocytotoxicity of the parent Pt complex in human cancer cell lines. Radiolabeled **Pt-succ-DFO-^68^Ga** (*t*_1/2_ = 68 min, positron emitter) was readily prepared under mild conditions and was stable in the dark upon incubation with human serum. PET imaging of **Pt-succ-DFO-**^**68**^**Ga** in healthy mice revealed a promising biodistribution profile with rapid renal excretion and limited organ accumulation, implying that little off-target uptake is expected for this class of agents. Overall, this research provides the first *in vivo* imaging study of the whole-body distribution of a photoactivatable Pt(IV) azido anticancer complex and illustrates the potential of radionuclide imaging as a tool for the preclinical development of novel light-activated agents.

## Abstract 2

Radiolabeling of the photoactivatable Pt(IV) complex **Pt-succ-DFO-Ga** with the radioisotope ^68^Ga allows PET imaging of its *in vivo* biodistribution.

## Introduction

Inorganic compounds, typically in the form of metals or minerals, have been used in medicine for centuries, but it was the discovery of the antiproliferative platinum compound cisplatin in the 1960s that ignited considerable interest in the development of metal-based drugs in the following decades.^1^ However, this substantial research effort has led to only a few successful clinical agents so far, mainly because lack of targeting and consequent severe side effects have hampered the translation of novel metallodrugs toward clinical use.

Light-activated metallodrugs provide spatial and temporal control of their cytotoxic activity by using visible light irradiation to achieve selective activation in the target tissue. Photodynamic therapy (PDT) uses photosensitizers, which can be excited by visible light irradiation to a long-lived triplet state. In this state, they can interact with molecular oxygen to yield the highly reactive singlet oxygen, which is responsible for their photocytotoxicity.^2^ These agents are catalytic, as upon energy transfer to molecular oxygen the photosensitizer is de-excited to its ground state and available to perform another cycle. However, their intrinsic dependence on tissue oxygenation can be problematic in hypoxic tumors, which can become resistant to treatment.^3^

Alternatively, light-activated metallodrugs may undergo chemical reactions upon light irradiation (photoactivation) to form cytotoxic species. Photoactivatable azido Pt(IV) agents are stable in the dark but can be reduced to cytotoxic Pt(II) species by visible light irradiation with concomitant release of azidyl radicals.^4^ Although this mechanism of action is unavoidably noncatalytic, it is oxygen-independent and promising for treatment of hypoxic cancers. Prototype azido Pt(IV) agent (*trans*,*trans*,*trans*-[Pt(pyridine)_2_(N_3_)_2_(OH)_2_]) **Pt1** is photocytotoxic in a range of cancer cell lines, irrespective of their sensitivity to cisplatin, suggesting a different mechanism of action.^5−7^ Platinum(IV) azido agents have been previously functionalized at the axial hydroxido ligand position to introduce peptides and proteins as targeting vectors,^8−11^ as well as other cytotoxic agents for multitargeting action,^12^ all without disrupting their photoactivation ability.

Imaging is a powerful tool to understand the biological behavior of drugs in cells and *in vivo* and to select promising candidates for preclinical development toward clinical translation. For light-activated agents, imaging can also guide the development of treatment protocols by identifying the optimum time for light-irradiation of the target cells/tissue based on the accumulation of the phototherapeutics.

*In vitro* fluorescence imaging has been extensively used to map luminescent Ru(II) and Ir(III) photosensitizers in cancer cells.^13−16^ Metal specific techniques, such as LA-ICP-MS or XRF, can be used to determine the accumulation and intracellular localization of the metal center in cancer cells.^17,18^ Recently, we have utilized synchrotron-XRF to visualize platinum from **Pt1** and its coumarin derivative in prostate cancer cells.^19^ While these techniques can also be applied *ex vivo*, as was shown by Rompel who imaged cancer tissue from xenograft models treated with gallium^20^ and ruthenium^21^ metallodrugs, they do not allow imaging *in vivo*.

Radionuclide imaging provides an effective way to track the fate of a molecule *in vivo* by exploiting a radioactive label. This approach has been previously utilized for PDT agents based on porphyrins and other tetrapyrrole derivatives.^22^ Radiolabeling of a porphyrin derivative has been previously performed using PET radionuclides ^18^F or ^124^I,^23,24^ but most commonly, the tetrapyrrole unit was radiolabeled with a radiometal, either directly in the tetrapyrrole ring (e.g., ^68^Ga or ^64^Cu),^25−29^ or by introducing a chelating fragment into the structure, able to bind radiometals to generate a theranostic agent.^30,31^ To the best of our knowledge, no examples of radiolabeled photoactivated chemotherapeutic (PACT) agents have been reported in the literature so far.

Deferoxamine (DFO, desferrioxamine) is a siderophore used by bacteria to acquire iron from their surroundings. DFO is clinically utilized to treat iron overload^32^ but has also gained popularity as a chelator for the PET radiometal ^89^Zr.^33^ Owing to its similarity to Fe(III) in ionic radius, oxidation state, and coordination preferences, Ga(III) can also be coordinated efficiently by DFO, and ^68^Ga radiolabeling of DFO occurs in mild conditions.^34^ Interestingly, besides providing a way to incorporate a radiometal in photoactivatable Pt(IV) complexes, DFO also displays interesting biological properties: it has been previously investigated as an anticancer treatment^35^ and was recently found to enhance the cytotoxic activity of cisplatin and carboplatin when used in combination with these agents.^36,37^ DFO conjugates of Pt(IV) carboplatin prodrugs have also been recently evaluated for their cytotoxic activity in cancer cells but were found to be less effective than either DFO or carboplatin alone.^38^

Here, we describe the synthesis of **Pt-succ-DFO**, a novel light-activated Pt(IV)-azido complex with a pendant DFO for radiometal chelation, and its corresponding heterobimetallic derivatives obtained by complexation with Fe(III) and Ga(III). We then report the photophysical properties and phototherapeutic potential of these agents in comparison with parent complex **Pt1**, to evaluate the effect of the axial succ-DFO ligand and its complexation with Ga(III) or Fe(III). Finally, we utilize the ^68^Ga-labeled derivative, **Pt-succ-DFO-**^**68**^**Ga**, to determine the serum stability of **Pt-succ-DFO-Ga** and its *in vivo* biodistribution in healthy animals.

## Experimental Section

General experimental considerations are included in the SI.

### Synthesis

*Caution!* While no problems were encountered during this work, heavy metal azides are known to be shock sensitive detonators, and extra care should be taken during handling.

Synthesis and handling of all photoactivatable complexes were performed in the dark with minimal light exposure to avoid photodecomposition.

**Pt1** and its succinate derivative **Pt-succ** were synthesized according to published procedures.^7,39^

#### Pt-succ-DFO

**Pt-succ** (15 mg, 0.026 mmol) was dissolved in DMF (0.5 mL), and TBTU (9.3 mg, 1.2 molar equiv) was added to the solution, followed by DIPEA (5.5 μL, 1.2 mol equiv). After stirring for 10 min, a suspension of DFO mesylate (17.3 mg, 0.026 mmol) and DIPEA (5.5 μL, 1.2 equiv) in DMF (0.5 mL) was added. The resulting mixture was stirred at room temperature overnight. The solvent was evaporated under reduced pressure, and the yellow-brown residue was purified on a Biotage Isolera system using a Biotage Sfär C18 12 g cartridge and a water/acetonitrile gradient. Lyophilization of the appropriate fractions resulted in 14 mg of a pale yellow solid (47.8% yield). The identity and purity of the complex were confirmed by ^1^H and ^13^C NMR, reverse-phase HPLC, and HR-MS.

HR-MS [C_39_H_62_N_14_O_12_Pt]: *m*/*z* [M + H]^+^ calcd 1114.4398 found 1114.4390, [M + Na]^+^ calcd 1136.4217 found 1136.4209.

^1^H NMR (500 MHz, *d*_4_-MeOD) *δH* = 8.92 (4 H, dd, ^3^*J*_1H1H_ = 5.5 Hz, ^3^*J*_195Pt1H_ = 26 Hz, H*o*), 8.26 (2H, t, ^3^*J*_1H1H_ = 7 Hz, Hp), 7.80 (4H, t, ^3^*J*_1H1H_ = 7 Hz, Hm), 3.60 (6H, s, C**H**_2_CH_2_CH_2_CH_2_CH_2_NH), 3.18 (6H, m, CH_2_CH_2_CH_2_CH_2_C**H**_2_NH), 2.78 (4H, t, COCH_2_C**H**_2_CONH from DFO), 2.64 (2H, t, COC**H**_**2**_CH_2_CONH from succinate), 2.44–2.47 (2H+4H,overlapping triplets COCH_2_C**H**_2_CONH from succinate and DFO, respectively), 2.11 (3H, s, CH_3_), 1.64 (6H, m, CH_2_C**H**_2_CH_2_CH_2_CH_2_NH), 1.53 (6H, m, CH_2_CH_2_CH_2_C**H**_2_CH_2_NH), 1.35 (6H, m, CH_2_CH_2_C**H**_2_CH_2_CH_2_NH).

^13^C NMR (125 MHz, *d*_4_-MeOD) *δC* = 177.06 (PtO**C**O); 173.52, 173.34, 173.08 (**C**ONH); 172.12 (**C**OCH_3_); 149.53 (C*o* pyridine); 141.66 (C*p* pyridine); 126.13 (C*m* pyridine), 38.96, 38.88, 31.7, 31.58, 30.07, 28.57, 28.54, 27.51, 25.93, 23.55, 23.50, 23.48 (methylene carbons); 18.84 (CO**CH**_**3**_).

#### Pt-succ-DFO-Ga

**Pt-succ-DFO** (4.4 mg, 1 molar equiv) was dissolved in 0.5 mL of aqueous ammonium acetate (40 mM in water), and the solution was combined with a solution of Ga(NO_3_)_3_·8H_2_O (2 mg, 1.3 molar equiv) in water (0.5 mL). The reaction mixture was stirred at room temperature for 1 h, after which it was loaded onto a C18 Sep Pak plus short cartridge (Waters) preconditioned with water. This was first washed with water to eliminate residual metal salts and then eluted with acetonitrile to obtain the final pure product. When the acetonitrile had evaporated, water was added to the sample followed by lyophilization to give 3.1 mg of a pale yellow product (66.5% yield). The identity and purity of the new heterobimetallic complex were confirmed by HR-MS and reverse-phase HPLC.

HR-MS [C_39_H_59_N_14_O_12_ GaPt]: [M + Na]^+^ calcd 1203.3232 found 1203.3244.

#### Pt-succ-DFO-Fe

**Pt-succ-DFO** (3.9 mg, 1 molar equiv) was dissolved in 0.5 mL of aqueous ammonium acetate (40 mM in water) and combined with a solution of Fe(NO_3_)_3_·9H_2_O (1.9 mg, 1.3 molar equiv) in water (0.5 mL) to yield a red solution. This mixture was stirred at room temperature for 1 h, after which it was purified as described for **Pt-succ-DFO-Ga**. When the acetonitrile had evaporated, water was added to the sample followed by lyophilization to give 3.1 mg of a dark red product (65.4% yield) The identity and purity of the new heterobimetallic complex were confirmed by HR-MS and reverse-phase HPLC.

HR-MS [C_39_H_59_N_14_O_12_ FePt]: [M + Na]^+^ calcd. 1189.3330 found 1189.3349

#### Photostability and Photodecomposition Studies

Solutions of **Pt-succ-DFO** (50 μM), **Pt-succ-DFO-Fe** (40 μM), and **Pt-succ-DFO-Ga** (40 μM) were prepared in 5% DMSO and 95% water (v/v). For dark stability studies, the UV–vis spectrum was monitored over time while keeping the solution in the dark. For photodecomposition studies, the UV–vis spectrum was measured at the same time points following irradiation (λ = 420 nm, 4.8 mW/cm^2^) with an LZC-ICH2 photoreactor (Luzchem Research Inc.) equipped with a temperature controller and 8 Luzchem LZC-420 lamps without light filtration.

#### Half-Maximal Inhibitory Concentration Determination

A2780 or A549 cells (10^4^ in 150 μL of medium) were seeded in each well of a 96-well plate and incubated at 310 K for 48 h. After this time, the supernatant was removed by aspiration, and cells were treated with six nominal concentrations (0.2, 2, 20, 50, 100, 200 μM) of **Pt-succ-DFO**, **Pt-succ-DFO-Fe**, and **Pt-succ-DFO-Ga** and their precursors **Pt1** and DFO, prepared in phenol red-free medium from a nominal 200 μM stock solution (containing 5% DMSO to aid solubility) in the dark. Exact concentrations of Pt stock solutions were determined retrospectively by ICP-OES using a PerkinElmer Optima 5300DV ICP-OES. Non-irradiated cells were incubated in the dark for 2 h, whereas irradiated cells were incubated for 1 h in the dark followed by 1 h irradiation using a 465 nm LED light source (4.8 mW/cm^2^). After light exposure, the Pt-containing supernatant was removed by aspiration, cells were washed with phosphate buffered saline (200 μL per well), and fresh medium (Pt-free) was added (200 μL per well). Cells were allowed a further 24 h recovery time in drug-free medium. Cells were fixed by addition of 50 μL of a 50% trichloroacetic acid solution (1 h, 277 K), and cell viability was determined using the sulforhodamine B assay.^40^ Half-maximal inhibitory concentrations (IC_50_) were determined by calculating cell survival relative to that of untreated controls. Each experiment was carried out as two independent experiments, each with biological triplicate samples (duplicate of triplicate). Mean and standard deviation values are reported.

#### Hemolysis

Fresh equine blood was centrifuged (10 min, 1000 g), the supernatant was removed, and the harvested erythrocytes were washed three times with PBS and then resuspended to a 5% erythrocyte concentration in PBS. **Pt-succ-DFO**, **Pt-succ-DFO-Fe**, and **Pt-succ-DFO-Ga** were dissolved in PBS in a 0.5–256 μg/mL serial dilution range. The resulting solutions (100 μL) were added to the suspended erythrocytes (100 μL) in 96-well round-bottom plates at 37 °C for 1 h without agitation. Controls included PBS and 1% Triton X-100 as 0% and 100% hemolysis, respectively. Each measurement was performed in triplicate. Hemolytic concentrations were determined to be >256 μg/mL (*ca*. 230 μM) for all compounds.

#### Radiolabeling

160 μL of a 315 μM solution of **Pt-succ-DFO** in water was mixed with 40 μL of a 1 M ammonium acetate solution. ^68^Ga was eluted from a ^68^Ge/^68^Ga-generator (Eckert & Ziegler) with 5 mL of 0.1 M HCl in 0.5 mL fractions, and their activity was measured on a dose calibrator (Capintec). A 200 μL aliquot of the highest activity fraction (80 MBq) was added to the buffered ligand, the solution was mixed by vortexing, and the pH was measured as 6. After a 10 min incubation, the radiolabeling was verified using radio-HPLC, which confirmed quantitative radiolabeling at a molar activity of 1585 MBq/μmol. A sham radiolabeling experiment in which ^68^Ga from the highest activity fraction was mixed with buffer only was also analyzed by HPLC to provide a profile for “free”, unchelated ^68^Ga. The radio-HPLC analysis was repeated on the same **Pt-succ-DFO-**^**68**^**Ga** batch 1 h later to verify stability toward radiation-mediated decomposition confirming that, at the molar activity reached, the complex is not susceptible to radiolysis.

#### Partition Coefficient Determination

^68^Ga-radiolabeling of **Pt-succ-DFO** was performed and verified, as described above. Then, an aliquot (10 μL) of the radiolabeling mixture was added to three vials containing a pre-equilibrated mixture of octanol/water (500/490 μL). The mixtures were vortexed and then shaken for 30 min before separation of the two phases by centrifugation (4000 rpm, 3 min). The activity in aliquots of each phase (20 μL aqueous phase, 100 μL octanol phase) was measured in the gamma-counter and corrected for the different volumes sampled.

#### Stability in Human Serum

**Pt-succ-DFO** was radiolabeled as previously described. Then 50 μL of the radiolabeling mixture was added to 150 μL of human serum from a healthy donor and incubated at 37 °C in the dark to evaluate the stability of the radiolabeled agent in serum. After 1 h, 200 μL of acetonitrile was added to precipitate the serum proteins, the vial was centrifuged, and the supernatant was removed. Measurement of protein pellet and supernatant ^68^Ga activity confirmed that only negligible (<10%) activity was associated with the proteins. The supernatant fraction was then evaporated under a stream of nitrogen to remove the acetonitrile, before redissolution in water and injection in the HPLC.

#### *In Vivo* Imaging and Biodistribution

All *in vivo* experiments were carried out in accordance with British Home Office regulations governing animal experimentation and complied with guidelines on responsibility in the use of animals in bioscience research of the U.K. Research Councils and Medical Research Charities, under U.K. Home Office projects and personal licenses.

Dynamic PET scanning was performed using a nanoPET/CT (Mediso Medical Imaging Systems).^41^ 4 female balb/C mice (7–9 weeks) were anesthetized with isoflurane (O_2_ flow rate of 1.0–1.5 L/min and isoflurane levels of 2–2.5%), cannulated at the tail vein using a catheter (27G), and placed on the scanner bed. The bed was heated to 37 °C by internal air flow to keep the animal at normal body temperature, and the respiration rate was monitored throughout scanning. A semicircular CT scan (55 kVp X-ray source, 600 ms exposure time in 180 projections) was performed, followed by a 120-min PET scan (1:5 coincidence mode; 5 ns coincidence time window). **Pt-succ-DFO-**^**68**^**Ga** (*ca*. 4 MBq in 100 μL of saline) was injected at the start of the PET scan. PET and CT data sets were reconstructed using the Monte Carlo-based full 3D iterative algorithm Tera-Tomo (Mediso Medical Imaging Systems) with the following settings for PET reconstruction: 4 iterations, 6 subsets, 1–3 coincidence mode, voxel sized 0.4 mm (isotropic), energy window 400–600 keV with attenuation, and scatter correction and binned into several frames (5 × 2 min, 4 × 5 min, 6 × 15 min). All reconstructed data sets were analyzed using VivoQuant 1.21 software (inviCRO).

After scanning, the animals were sacrificed by neck dislocation while still anesthetized. *Ex vivo* biodistribution was performed by collecting the whole organ in the cases of tail, heart, lungs, liver, spleen, stomach, and kidneys. The tibia was used as representative of bone. Skin and fur were collected from the ears of the mouse, and muscle was taken from the hind limb. Only part of the liver and small and large intestine was collected. All organs were washed in water to eliminate residual blood, weighed, and measured using a Wallac gamma counter. Residual activity in the tail (due to any imperfections in *iv* injection) was subtracted from the total activity injected, and tail-corrected %ID was calculated for each organ. %ID/g was obtained by dividing the tail-corrected %ID of each organ by its weight.

## Results

### Synthesis and Characterization

**Scheme 1:**
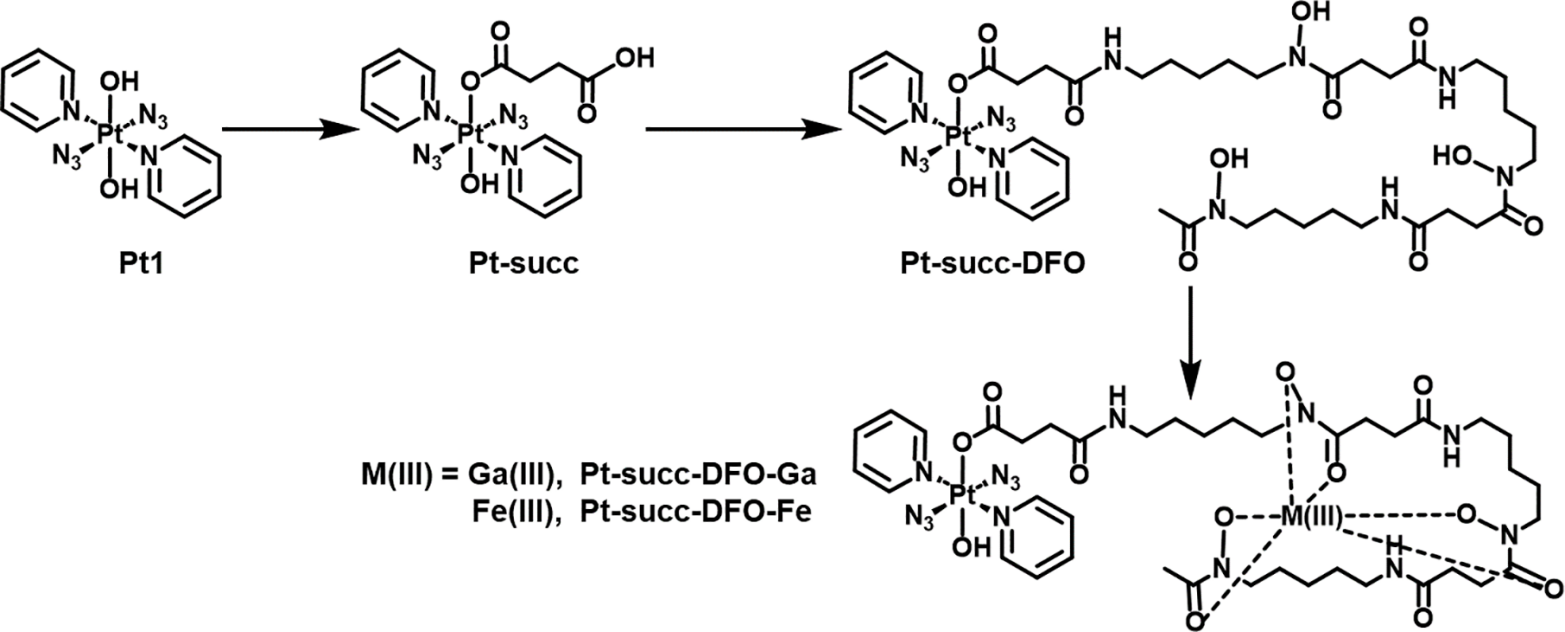
Synthetic Route to Deferoxamine Complex **Pt-succ-DFO** and Its Heterobimetallic Derivatives **Pt-succ-DFO-Ga** and **Pt-succ-DFO-Fe**

**Pt-succ-DFO** was synthesized in two steps from the prototype complex **Pt1** (Scheme 1) by first introducing succinate as an axial ligand following literature procedures^39^ and then coupling it with the free amino group of DFO, followed by flash chromatography purification. The identity and purity of the complex were verified by HR-MS and HPLC (Figure S1), and NMR data (Figure S2 and S3) were consistent with the proposed structure. ^1^H NMR, for example, showed the characteristic ^195^Pt satellite peaks for the doublet representing the pyridine ortho proton at 8.91 ppm.

**Pt-succ-DFO** was then reacted with a slight excess of a Ga(III) or Fe(III) salt in an aqueous ammonium acetate solution at pH 6, to obtain **Pt-succ-DFO-Ga** and **Pt-succ-DFO-Fe**, respectively. These heterobimetallic complexes were purified by solid-phase extraction on an SPE C18 cartridge, and their identity and purity were confirmed by HR-MS and HPLC (Figure S1).

### Photoactivation Properties

The UV–vis spectrum of **Pt-succ-DFO** (Figure S4) showed the same features as the parent complex **Pt1** including a mixed LMCT/interligand transition at 259 nm and the Pt ← N_3_ LMCT band with an absorbance maximum at 298 nm. The Fe(III)/Ga(III) derivatives displayed remarkably similar spectra, except that **Pt-succ-DFO-Fe** exhibited an additional third broad band at 415 nm, attributable to a Fe ← DFO LMCT transition,^42^ which accounted for the dark red color of this complex as opposed to the pale yellow of the other two. In the same spectral region, the three complexes also have a weak shoulder attributable to mixed ^1^LMCT/^1^IL transitions involving N_3_ and OH ligands as well as the Pt(IV) center, as previously observed for this family of complexes.^5^ Due to its low intensity, this band is distinguishable only at higher concentrations (Figure S5).

**Figure 1:**
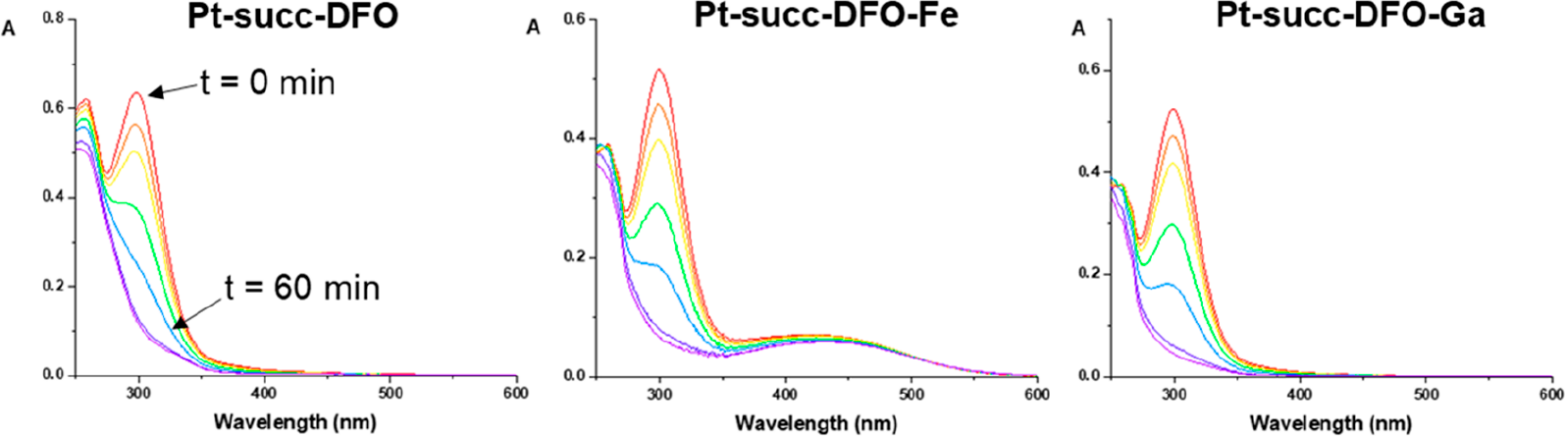
UV–vis spectra of **Pt-succ-DFO** (50 μM) and its Ga(III) and Fe(III) complexes (40 μM) upon 420 nm light irradiation, showing reduction in the intensity of the (Pt ←N_3_) LMCT band.

Photostability and photoactivation properties of the three complexes were studied by UV–vis spectroscopy in the dark and upon irradiation with indigo light (420 nm, 4.8 mW/cm^2^). All complexes showed excellent stability in the dark, as well as rapid photodecomposition upon irradiation (Figure 1), as indicated by the decrease in intensity of the LMCT band at 298 nm. No decrease in the intensity of the Fe ← DFO LMCT band was observed for **Pt-succ-DFO-Fe**. All three complexes showed similar photodecomposition kinetics (Figure S6).

**Figure 2:**
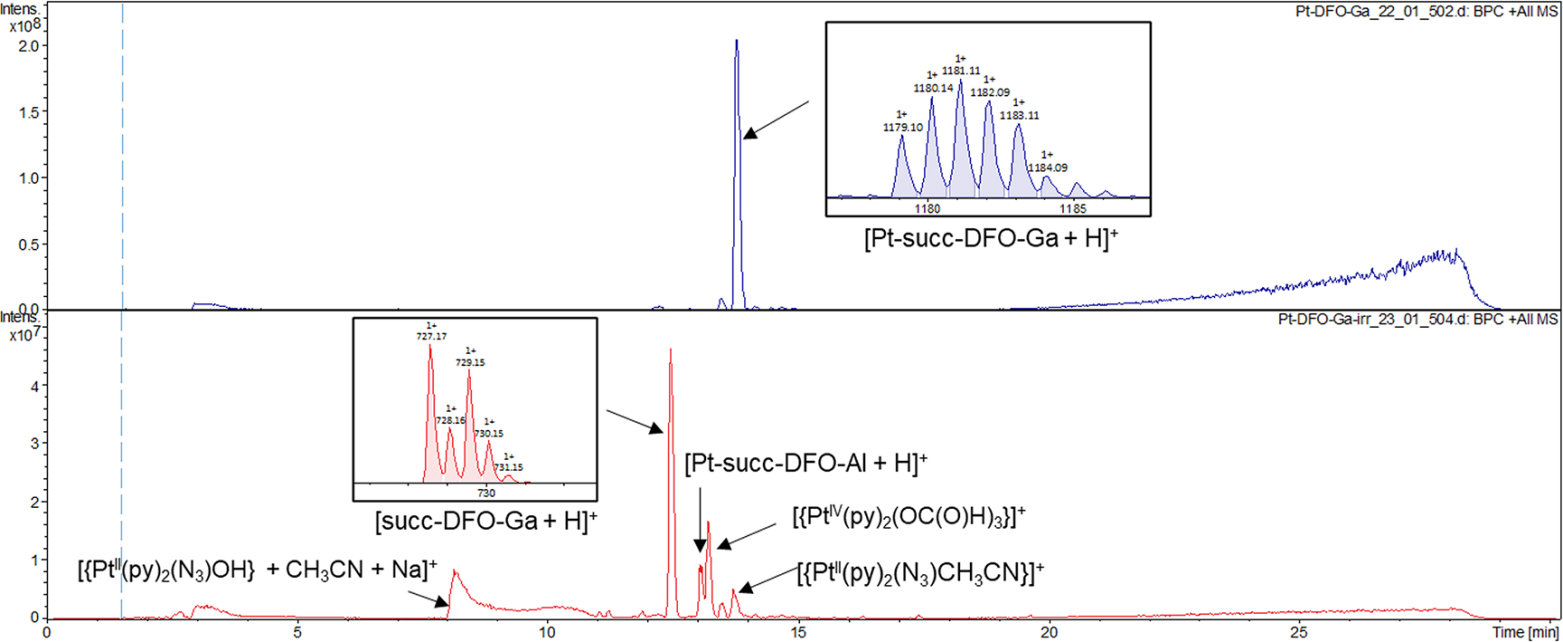
LC-MS analysis of **Pt-succ-DFO-Ga** before (top) and after (bottom) 2 h of irradiation with 420 nm light.

LC-MS analysis of the **Pt-succ-DFO**-**Ga** complex upon photoactivation (Figure 2) showed the released succinate-DFO-Ga axial ligand as the main peak, indicating that, similar to previous reports for other Pt(IV) diazido derivatives, these agents tend to lose both axial ligands upon light irradiation, pointing to a similar mechanism of photoactivation irrespective of the axial ligand.^8^ Other peaks were assigned to typical photoproducts of prototype complex **Pt1** (see Table S1 in the ESI for full assignment). Interestingly, although the peak of the intact complex had completely disappeared, a small peak corresponding to an aluminum complex (Pt-succ-DFO-Al) was observed, likely due to trace Al in the LC-MS instrument.

### *In Vitro* Biological Evaluation

The photocytotoxicity of **Pt-succ-DFO**, its heterobimetallic derivatives and their precursors **Pt1** and DFO was determined in the non-small cell human lung cancer cell line A549 and the human ovarian cancer cell line A2780 using the sulforhodamine B (SRB) assay. The typical photoactivation protocol was a 1 h incubation in the dark followed by a 1 h irradiation at 465 nm, while cytotoxicity in the absence of irradiation was determined for an incubation of 2 h in the dark. The three **Pt-succ-DFO** complexes exhibited similar behavior to **Pt1** with a significant increase in cytotoxicity observed upon irradiation in both cell lines, while no cytotoxicity was observed for DFO under any of the conditions tested (Table 1). Notably, the half-maximal inhibitory concentrations measured for **Pt-succ-DFO**, **Pt-succ-DFO-Ga**, and **Pt-succ-DFO-Fe** upon irradiation in each cell line were remarkably similar. On the other hand, **Pt1** exhibited higher photocytotoxicity in A2780 cells compared to its DFO derivatives.

**Table 1:** Half-Maximal Inhibitory Concentration (IC_50_) Values for **Pt-succ-DFO** and Its Ga(III) and Fe(III) Derivatives

	**IC_50_/μM**^a^			
	**A2780**		**A549**	
**Complex**	**Dark**	**Irrad.(465 nm)**	**Dark**	**Irrad.(465 nm)**
**Pt-succ-DFO**	>150	56 ± 8	>100	47 ± 3
**Pt-succ-DFO-Fe**	>50	28 ± 2	>200	40 ± 6
**Pt-succ-DFO-Ga**	>100	34 ± 2	>150	55 ± 7
**DFO**	>200	>200	>200	>200
**Pt1**	>100	11.4 ± 0.5	>100	52 ± 3

a Treatment conditions: a 2 h incubation (dark) or 1 h incubation + 1 h irradiation at λ = 465 nm (irradiation) followed by a 24 h recovery. Complex **Pt1** and DFO were also assessed for comparison. Cell survival was determined using the SRB assay. Data reported are the average and standard deviation of two independent experiments, each performed in triplicate.

Prior to *in vivo* testing of Pt-succ-DFO agents, hemolysis screening in equine erythrocytes was also performed to confirm that the intravenous injection of these agents would not result in toxicity. Neither **Pt1** nor its **Pt-succ-DFO** derivatives induced any hemolysis under the conditions tested (Experimental Section).

### ^68^Ga Radiolabeling of Pt-succ-DFO

**Figure 3:**
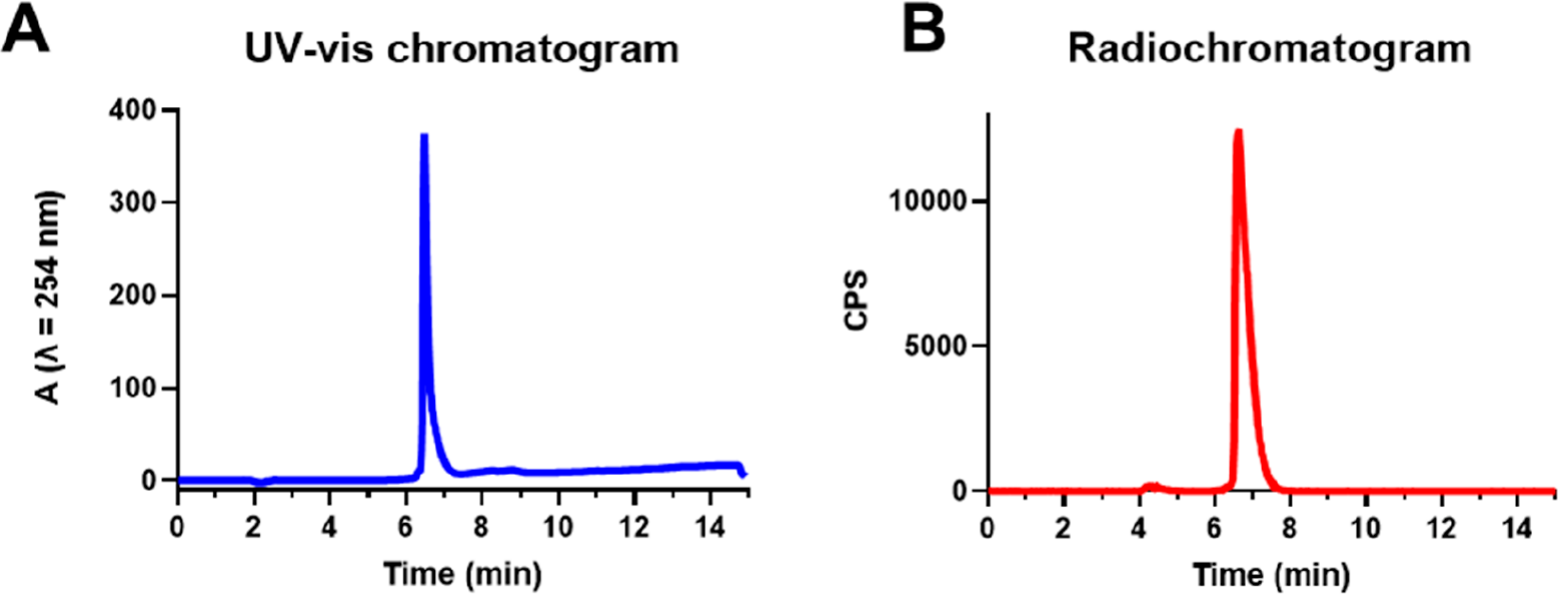
HPLC analysis of **Pt-succ-DFO-**^**68**^**Ga** confirms the presence of a single radiolabeled species in the radiochromatogram (B), corresponding to quantitative ^68^Ga incorporation into **Pt-succ-DFO** (UV–vis chromatogram, A). CPS = counts per second.

Radiolabeling of **Pt-succ-DFO** (126 μM) with ^68^Ga (80 MBq) from an Eckert & Ziegler ^68^Ge/^68^Ga generator was performed at ambient temperature in the same mild aqueous conditions utilized for the non-radioactive complexation reaction. The radiolabeling yielded a radiochemically pure **Pt-succ-DFO-**^**68**^**Ga** complex (Figure 3), without the need for further purification, as shown in the radio-HPLC (Figure 3) displaying a single peak at 6 min 36 s eluting just after the peak at 6 min 28 s attributable to excess unlabeled **Pt-succ-DFO**. The occupancy of Pt-succ-DFO molecules by ^68^Ga is < 0.002% at the molar activity achieved. In contrast, “free” unchelated gallium eluted with the solvent front under the same gradient conditions (Figure S7). No changes in the HPLC trace were observed within 1 h after radiolabeling, demonstrating that **Pt-succ-DFO-**^**68**^**Ga** is stable to radiolysis in the dark at the molar activity achieved (Figure S8). The water/octanol partition coefficient for **Pt-succ-DFO-**^**68**^**Ga** was measured using the shake-flask method yielding LogP = −2.52 ± 0.06, as expected for a complex that combines several hydrophilic components. Since similar retention times were measured for all Pt-succ-DFO complexes by reversed-phase HPLC, it can reasonably be assumed that **Pt-succ-DFO** and **Pt-succ-DFO-Fe** also have similar hydrophilicity.

Prior to performing *in vivo* experiments, the stability of **Pt-succ-DFO-**^**68**^**Ga** in serum was evaluated by radio-HPLC analysis of the radioactive agent upon 1 h of serum incubation and protein removal by precipitation. This showed a single radiolabeled peak on the chromatogram with the retention time of the **Pt-succ-DFO-**^**68**^**Ga** complex, confirming its stability in serum in the absence of irradiation (Figure S9).

### *In Vivo* Imaging and Biodistribution in Healthy Animals

**Figure 4:**
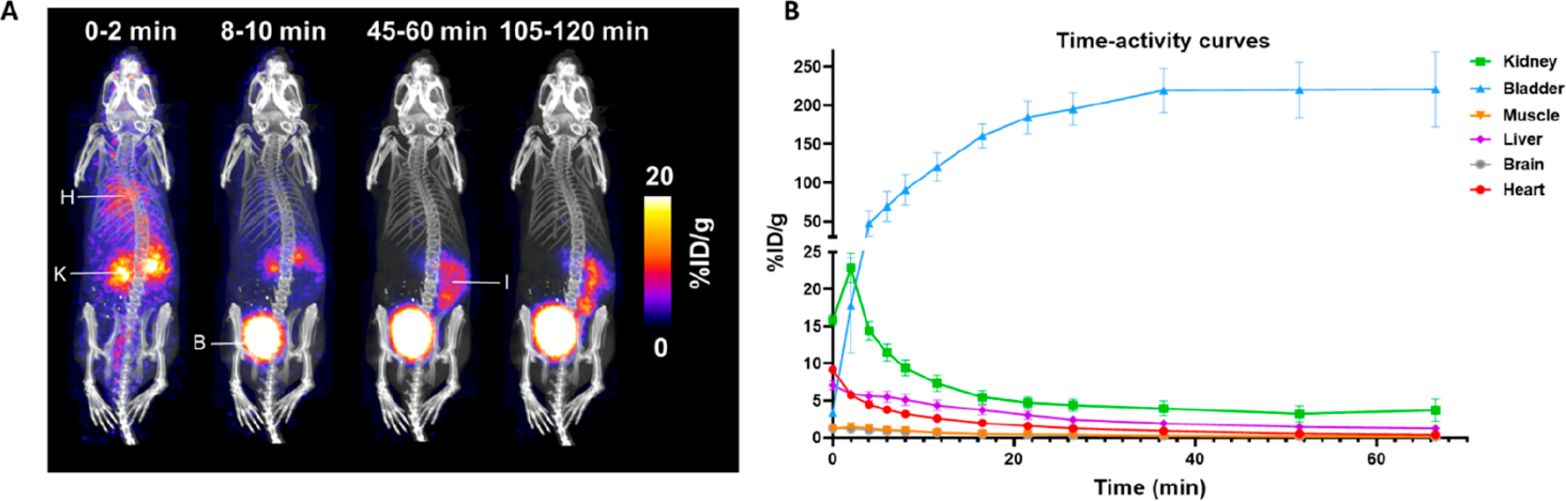
*In vivo* imaging of **Pt-succ-DFO-**^**68**^**Ga** in healthy mice. A) Exemplar Maximum Intensity Projections (MIPs) obtained by dynamic PET imaging, showing how biodistribution of the radiolabeled agent changes with time. Highlighted organs are H = heart, K = kidney, B = bladder, I = intestine. B) Image-derived time-activity curves for selected organs showing rapid renal excretion and low tissue accumulation (accumulation in muscle and brain is negligible). Data show average ± standard error.

The biodistribution of **Pt-succ-DFO-**^**68**^**Ga** was measured in healthy mice by using dynamic PET imaging (Figure 4, Figure S11). The PET images and resulting time-activity curves show that most of the **Pt-succ-DFO-**^**68**^**Ga** was quickly excreted through the kidneys into the urine with minimal accumulation in any tissue. A minor portion of the radiolabeled product was excreted via the hepatobiliary route, as was also confirmed by the *ex vivo* biodistribution performed at 2 h after injection where some accumulation in the gastrointestinal tract (particularly bile and small intestine) was visible, particularly for 2 of the 4 animals (Figure S10).

## Discussion

Radionuclide imaging has the potential to accelerate the preclinical development of new drugs by providing an efficient and robust way to evaluate their pharmacokinetic properties and biodistribution *in vivo*. In the case of phototherapeutics, including photosensitizers for PDT and photoactivatable agents, radionuclide imaging can also inform the development of phototherapeutic protocols that maximize treatment efficacy, by determining the time of maximum accumulation of the sensitizer at the target tissue after administration.

In this work, we have developed **Pt-succ-DFO**, a new photoactivatable Pt(IV) agent containing the conjugated iron chelator DFO, and investigated its *in vivo* behavior using dynamic ^68^Ga PET imaging. **Pt-succ-DFO** was readily synthesized and converted to new heterobimetallic complexes **Pt-succ-DFO-Fe** and **Pt-succ-DFO-Ga** by complexation of Fe(III) and Ga(III), respectively, under mild aqueous conditions.

The three complexes were photoactivatable upon irradiation with indigo light at 420 nm (corresponding to low-energy mixed ^1^LMCT/^1^IL transition^5^), as was apparent by changes in the UV–vis spectra, particularly in the progressive reduction of the Pt ← N_3_ LMCT band at 298 nm. No changes upon irradiation were observed for the Fe ← DFO LMCT band of **Pt-succ-DFO-Fe** (λ = 415 nm), suggesting that this transition is not involved in the photoactivation processes. LC-MS analysis of **Pt-succ-DFO-Ga** after irradiation revealed release of axial hydroxido and succ-DFO ligands and similar photodecomposition products as previously observed for precursor **Pt1**.^2,5^ Overall, these results suggest that the succ-DFO or succ-DFO-Ga(III)/Fe(III) fragment does not participate in the photodecomposition process but is simply released as a result.

Photocytotoxicity evaluation in cancer cells was conducted using blue light (λ = 465 nm) to provide a reasonable light penetration depth. All **Pt-succ-DFO** complexes showed light-mediated cytotoxicity, with similar IC_50_ values irrespective of whether the DFO moiety was coordinated to Fe(III) or Ga(III). This indicates that the coordination of DFO to Ga(III)/Fe(III) has little effect on the biological activity of **Pt-succ-DFO** complexes. On the other hand, the parent complex **Pt1** showed a different photocytotoxicity profile, with similar IC_50_ values for A549 cells, but markedly lower IC_50_ for A2780 cells. The differences in biological activity are likely to be associated with the change in the size and physicochemical properties of **Pt-succ-DFO** derivatives compared to **Pt1**, which could influence, for example, the extent of cancer cell accumulation as previously shown for other **Pt1** derivatives.^19^ Alternatively, a partially antagonistic interaction between **Pt1** photoproducts and the released **succ-DFO** moiety in A2780 cells could also be partly responsible for the lower photocytotoxicity observed for Pt-succ-DFO compounds compared to the parent complex **Pt1**.

Radiolabeling of **Pt-succ-DFO** with ^68^Ga was quantitative under mild reaction conditions, yielding the first radiolabeled photoactivatable Pt(IV) agent **Pt-succ-DFO-**^**68**^**Ga**, which was stable to radiolysis at the relatively low molar activity achieved. This was important to verify because some light-activated metallodrugs have previously been reported to be also activated by X-ray irradiation.^43−45^

Radiolabeling of **Pt-succ-DFO** confirmed that this complex is stable in serum in the absence of light irradiation. This is particularly important since, based on experiments with relevant biomolecules (such as the biological reductant glutathione) it was proposed that, unlike traditional Pt(IV) prodrugs, photoactivatable Pt(IV) agents should be stable to reduction in the dark in biological systems.^39^ This work demonstrates that **Pt-succ-DFO-Ga** is stable toward reduction in human serum.

Dynamic PET imaging of **Pt-succ-DFO-**^**68**^**Ga** revealed a promising pharmacokinetic profile with rapid renal excretion and low accumulation in any tissue. This suggests that, if a targeting moiety can be introduced, for example by attaching a cancer-targeting peptide as a second axial ligand,^12^ little off-target uptake is expected in other tissues. A natural limitation of this study is that ^68^Ga PET imaging reveals only the fate of ^68^Ga irrespective of whether the **Pt-succ-DFO-**^**68**^**Ga** complex is still intact. Further studies could investigate the *in vivo* stability of the complex, for example, by analyzing organ distribution of Pt via ICP-MS on digested tissues and comparing with data obtained for ^68^Ga by gamma counting. On the other hand, the serum stability demonstrated by the **Pt-succ-DFO-**^**68**^**Ga** suggests that the complex should be stable in blood circulation in the absence of photoirradiation. Notably, the ^68^Ga biodistribution observed by dynamic PET imaging is typical of a small hydrophilic molecule such as **Pt-succ-DFO-**^**68**^**Ga**, rather than of unchelated Ga which displays slower clearance and a more diffuse distribution.^46^

Beyond cancer phototherapy, another potential application of **Pt-succ-DFO-**^**68**^**Ga** is in the field of photoactivated antibiotics, exploiting the ability of several bacteria (*e.g*. *Pseudomonas aeruginosa*, *Staphylococcus aureus*) to take up iron and gallium complexes of the siderophore DFO.^47^ A similar approach has been previously utilized for siderophore-conjugates of established antibiotics.^48^

In either case, radiolabeling of the DFO fragment with ^68^Ga would enable an imaging-guided treatment approach by tracking accumulation of phototherapeutic prodrugs at the desired target site, allowing design of photoactivation protocols that maximize therapeutic efficacy. Although blue-light activation limits the application of these agents due to poor tissue penetration, they could be useful for diseases readily accessible to light such as premalignant lesions in the lungs or lung infections that can be irradiated using an endoscopic light.

## Conclusions

We have developed a novel photoactivatable Pt(IV) azido agent that can incorporate the PET radionuclide ^68^Ga. We utilized the resulting radiolabeled **Pt-succ-DFO-**^**68**^**Ga** complex to image, for the first time, the *in vivo* behavior of this promising class of platinum phototherapeutics. **Pt-succ-DFO-**^**68**^**Ga** was stable in serum and displayed a promising biodistribution profile with rapid renal excretion and low tissue accumulation, encouraging further preclinical development of photoactivatable Pt(IV) azido agents, particularly those conjugated to cancer-targeting molecules.

More generally, this work serves as a proof of concept to demonstrate the utility of radiolabeling and radionuclide imaging to investigate the properties of photoactivatable agents, thus facilitating their preclinical development and ultimately their clinical translation.

## Supporting Information Available

Additional experimental details, full assignment of LC-MS peaks for **Pt-succ-DFO-Ga**, characterization data (including HPLC chromatograms, ^1^H and ^13^C NMR spectra, UV–vis spectra of the complexes without irradiation), additional radiolabeling data, *ex vivo* biodistribution, and additional PET MIP (PDF)
