# Supplementary material for: Radiometal-Labeled Photoactivatable Pt(IV) Anticancer Complex for Theranostic Phototherapy
Source: Inorg Chem. 2023 Aug 29;62(50):20745–53. doi: 10.1021/acs.inorgchem.3c02245 (PMC10731635; doi:10.1021/acs.inorgchem.3c02245)
Supplement: Supplementary file 1 — ic3c02245_si_001.pdf [file ic3c02245_si_001.pdf]

Radiometal-labeled photoactivatable Pt(IV) anticancer complex for theranostic phototherapy

Cinzia Imberti, * [a] Jamie Lok, [a] James P. C. Coverdale,[b] Oliver W. L. Carter,[a] Millie E. Fry,[b] Miles L. Postings,[a] Jana Kim,[c] George Firth,[c] Philip J. Blower,[c] Peter J. Sadler. *[a]

[a] Department of Chemistry, University of Warwick, Coventry, CV4 7AL, UK

[b] School of Pharmacy, Institute of Clinical Sciences, University of Birmingham, Birmingham, B15 2TT, UK

[c] School of Biomedical Engineering & Imaging Sciences, King's College London, St Thomas' Hospital, London, SE1 7EH, UK

Corresponding Authors

Cinzia Imberti – Email: Cinzia.imberti@warwick.ac.uk

Peter J. Sadler – Email: P.J.Sadler@warwick.ac.uk

Content

Further Experimental Details

Table S1. Assignments of LC-MS chromatogram peaks for **Pt-succ-DFO-Ga**

Figure S1 HPLC UV-vis chromatogram of **Pt-DFO** and its heterobimetallic derivatives

Figure S2 ^{1}H-NMR of complex **Pt-succ-DFO**

Figure S3 ^{13}C-NMR of complex **Pt-succ-DFO**

Figure S4 UV-vis spectra of **Pt-succ-DFO** and its heterobimetallic derivatives in the dark

Figure S5 UV-vis spectrum of **Pt-succ-DFO** highlighting ^{1}LMCT/^{1}IL band

Figure S6 Photodecomposition kinetics for the three **Pt-succ-DFO** complexes

Figure S7 Radio-chromatogram of unchelated $^{68}Ga^{3+}$

Figure S8 HPLC analysis of **Pt-succ-DFO-^{68}Ga** 1 h after radiolabeling

Figure S9 Radio-HPLC analysis of **Pt-succ-DFO-^{68}Ga** after 1 h incubation in human serum

Figure S10 *Ex vivo* biodistribution of **Pt-succ-DFO-^{68}Ga**

Figure S11 MIP images of **Pt-succ-DFO-^{68}Ga** biodistribution at different time points

Experimental Section

Materials and Methods

K_2PtCl_4 was purchased from Acros Organics, pyridine from Fisher Scientific and TBTU from Fluka Chemicals. All other chemicals were obtained from Sigma Aldrich, unless otherwise specified, and used without further purification.
NMR spectra were recorded on a Bruker Avance III HD 500 MHz (^{1}H) spectrometer at 298 K. Chemical shifts were referenced and calibrated to CD_3OD residual solvent peak. High-resolution mass spectrometry (HRMS) was recorded with a Bruker maXis plus QTOF.
UV-visible absorption spectra were recorded on a Varian Cary 300 UV-visible spectrometer in a quartz cuvette and solvent used to measure baseline. The spectral width was 200–600 nm and the bandwidth was 1.0 nm, the scan rate was set to 600 nm/min. A Luzchem LZC-ICH2 photoreactor with 420 nm 200 fc lamps was used to irradiate the samples.
Reverse-phase HPLC, radio-HPLC and LC-MS analysis was performed using water with 0.1% formic acid as mobile phase A and acetonitrile with 0.1% formic acid as mobile phase B. Flow rate was set at 1 mL/min. Solvent gradients used were:
Gradient 1: 0-5 min 95% A, 5-30 min from 95% to 15% A, 25-27 min from 20% to 95% A, 27-30 min 95% A.
Gradient 2: 0-2 min 95% A, 2-11 min from 95% A to 5% A, 11-12 min 5% A, 12-15 min from 5% A to 95% A.
Purity checks of all final complexes were performed on an Agilent 1100 HPLC instrument using an Agilent Zorbax eclipse Plus C18 column (4.6 x 250 mm, 5 µm) and gradient 1. LC-MS was carried out using the same Zorbax eclipse Plus C18 column on an Agilent 1260 HPLC system connected to a Bruker Amazon X Iontrap MS instrument using gradient 1.
Radio-HPLC was performed on an Agilent 1200 LC system with UV detection at 214 or 254 nm coupled to a LabLogic Flow-Count detector with a sodium iodide probe (B-FC-3200), using an Agilent Eclipse XDB-C18 column (4.6 x 150 mm, 5 µm) using gradient 2.
^{68}Ga was obtained from a GalliaPharm Eckert & Ziegler (E&Z) generator eluted with 0.1 M clinical grade HCl (E&Z).
Handling of radioactivity was performed in a lead workstation to minimize exposure, and conducted according to standard safety regulations.

Cell Culture

A2780 ovarian cancer cells and A549 lung cancer cells were purchased from Public Health England and tested for mycoplasma-free status at 6-monthly intervals. Cells were maintained using DMEM culture medium supplemented with 10% FBS and 1% penicillin/streptomycin antibiotics, in a 37 °C incubator with a 5% CO_2 humidified atmosphere. Cells were passaged upon reaching 80% confluence.

SRB assay

Fixed cells were washed with water and stained with 50 µL of 0.4% SRB solution in 1% acetic acid for 1 h, followed by washing with 1% acetic acid. Plates were dried thoroughly and 100 µL of 10 mM Tris Base was added. After 1 h incubation, absorbance at 492 nm was measured using an Omega microplate reader. Data were analyzed using Microsoft Excel and GraphPad Prism 9 (version 9.5.1) for MacOS.

ICP-OES

Calibration standards (Pt, 10-1000 ppb) were freshly prepared from 1000 µg/mL CRM material (Inorganic Ventures) in 3.6% v/v nitric acid (prepared using milliQ water) and spiked with standard addition of sodium chloride (99.9999% trace metal basis) to match salinity of cell media. 100 µM samples of complexes in media were diluted 50-fold in 3.6% v/v nitric acid prior to analysis. Samples were analyzed using a Perkin Elmer Optima 5300DV ICP-OES spectrophotometer and the data were processed using WinLab32 for Windows.

Table S1. Assignments of LC-MS chromatogram peaks for **Pt-succ-DFO-Ga** in the dark or upon 2h irradiation (465 nm). Major peaks are coloured in light blue.

Experiment	Species	m/z	Retention time (min)[a]
**Dark**	$[DFO\text{-}Fe + H]^+$	619.3	13.4
	$[Pt\text{-}succ\text{-}DFO\text{-}Ga + H]^+$	1181.1	13.8
**Light**	$[\{Pt^{II}(py)_2(N_3)OH\} + CH_3CN + Na]^+$	475.8	8.1
	$[H\text{-}succ\text{-}DFO\text{-}Ga + H]^+$	727.2	12.5
	$[Pt\text{-}succ\text{-}DFO\text{-}Al + 2H]^{2+}$	569.5	13.0
	$[\{Pt^{IV}(py)_2(OC(O)H)_3\}]^+$	487.9	13.2
	$[DFO\text{-}Fe + H]^+$	619.3	13.5
	$[\{Pt^{II}(py)_2(N_3)(CH_3CN)\}]^+$	435.9	13.7

[a] HPLC chromatogram in **Figure 2**.

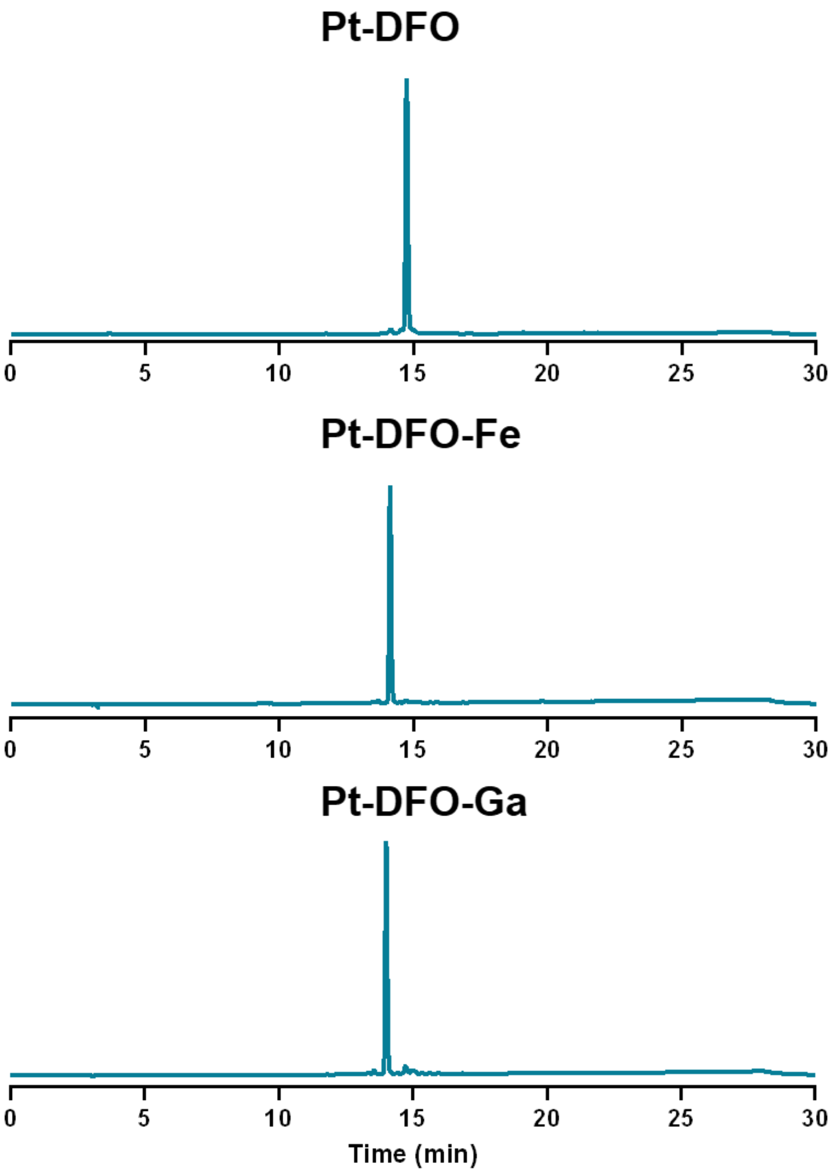

Figure S1. HPLC UV-vis chromatograms of **Pt-succ-DFO** (top) and its heterobimetallic Fe(III) (middle) and Ga(III) (bottom) derivatives (detection wavelength 254 nm), gradient 1.

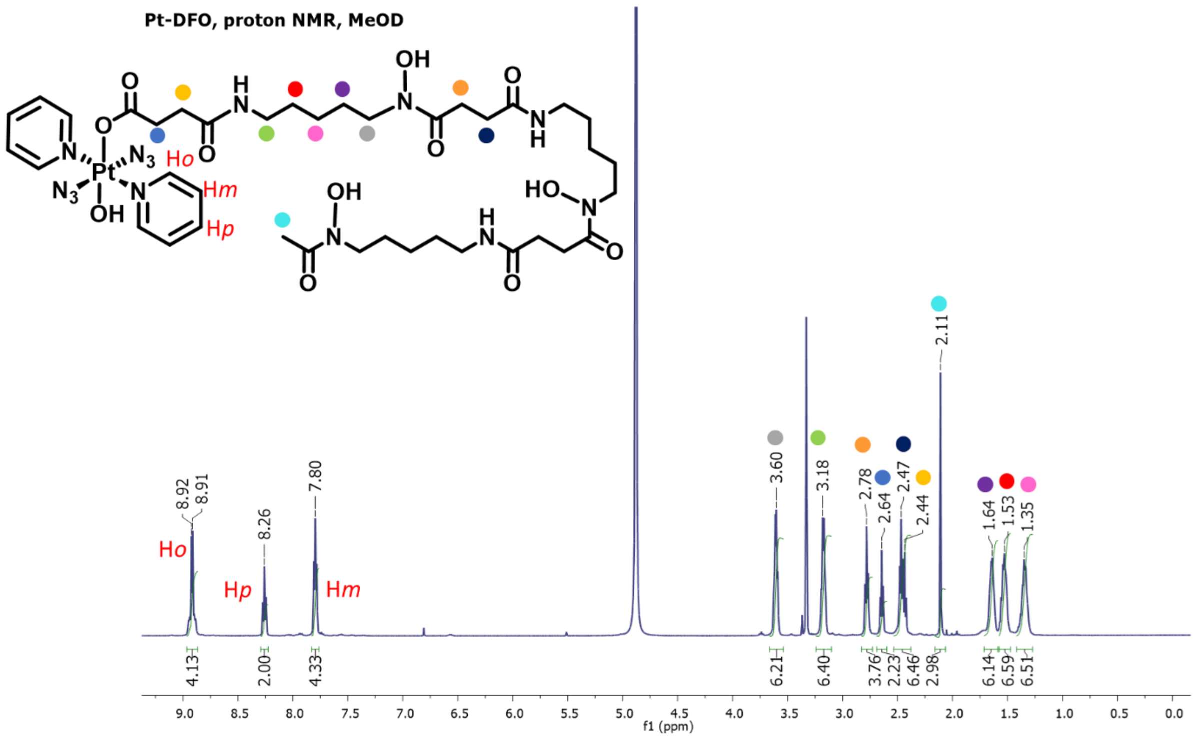

Figure S2. ^{1}H-NMR spectrum of complex **Pt-succ-DFO** in CD_3OD.

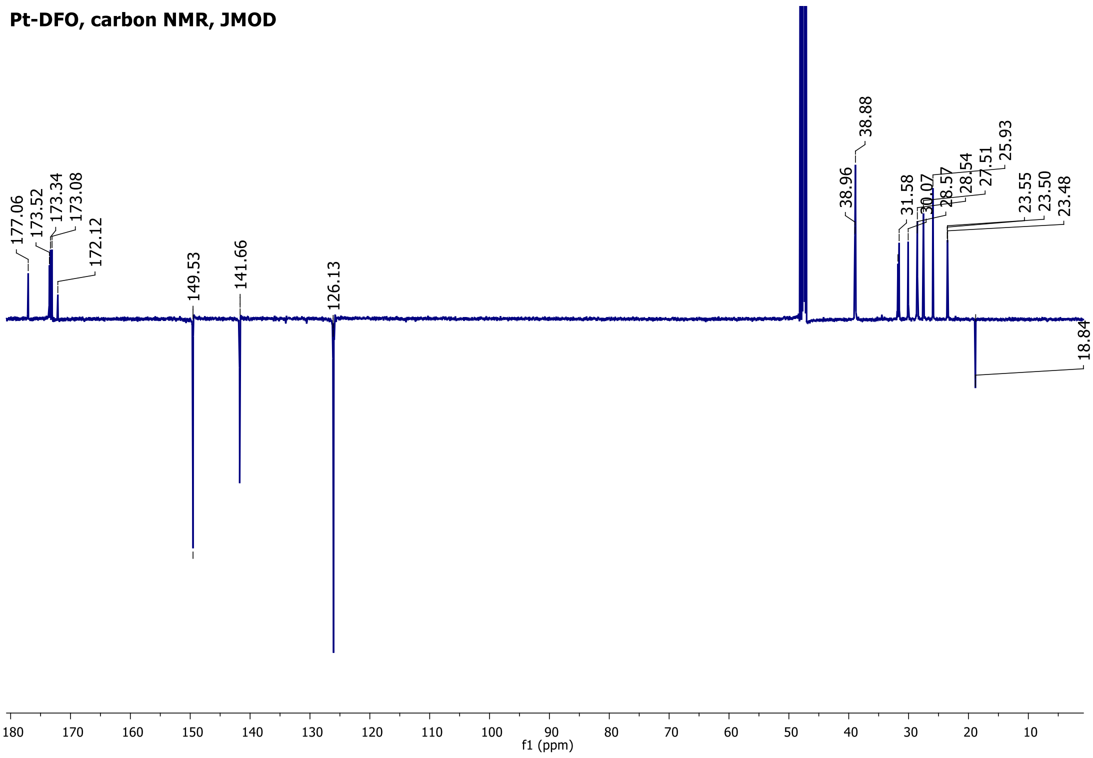

Figure S3. ^{13}C J-modulated spin-echo NMR spectrum of complex **Pt-succ-DFO** in CD_3OD (peak assignment in manuscript).

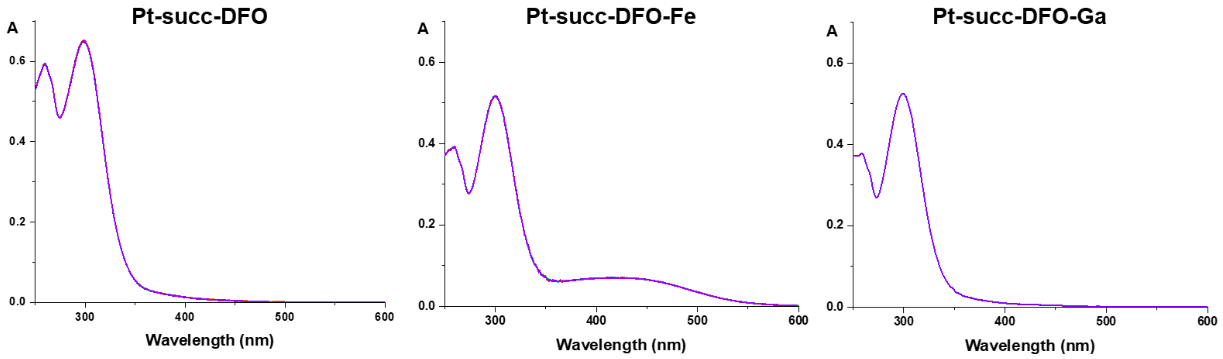

Figure S4. UV-vis spectra of **Pt-succ-DFO** and its heterobimetallic Fe(III) and Ga(III) derivatives in the dark (solvent: 5% DMSO in water).

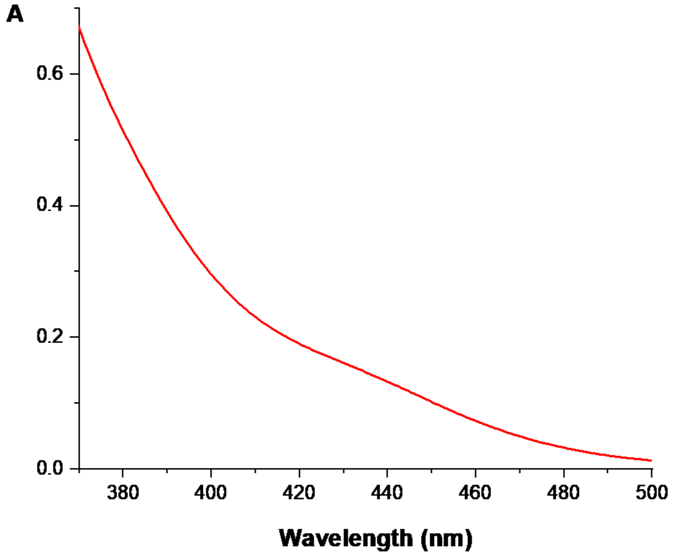

Figure S5. UV-vis spectrum of **Pt-succ-DFO** at high concentration (900 µM) highlighting the weak band corresponding to the predicted ^{1}LMCT/^{1}IL transition (shoulder at 430 nm; ε *ca.*, 170 M^{-1} cm^{-1}).

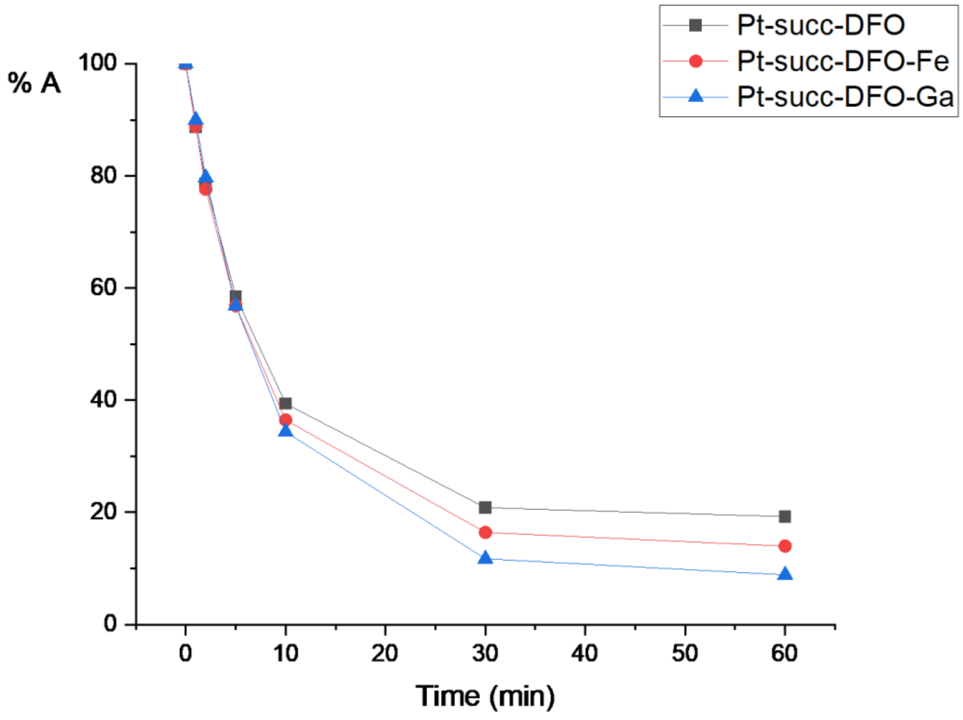

Figure S6. Photodecomposition kinetics for the three **Pt-succ-DFO** complexes determined by UV-vis spectroscopy (irradiation at 420 nm, detection at 298 nm).

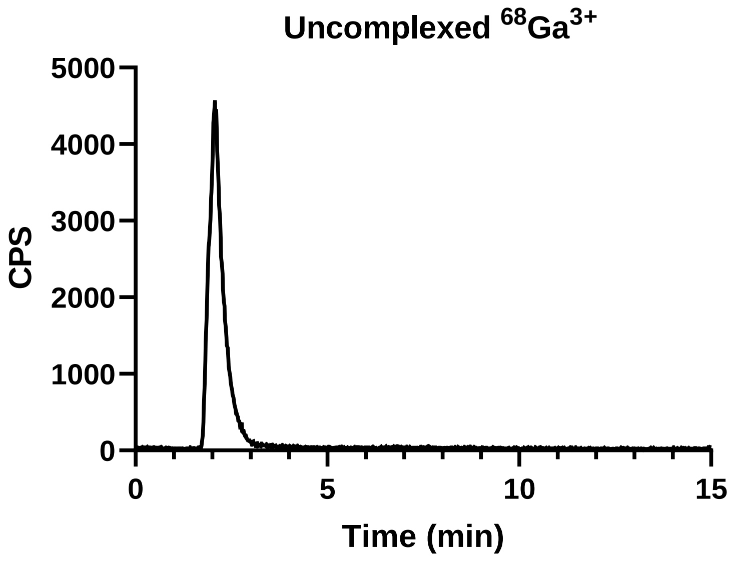

Figure S7. Radio-chromatogram of unchelated $^{68}Ga^{3+}$.

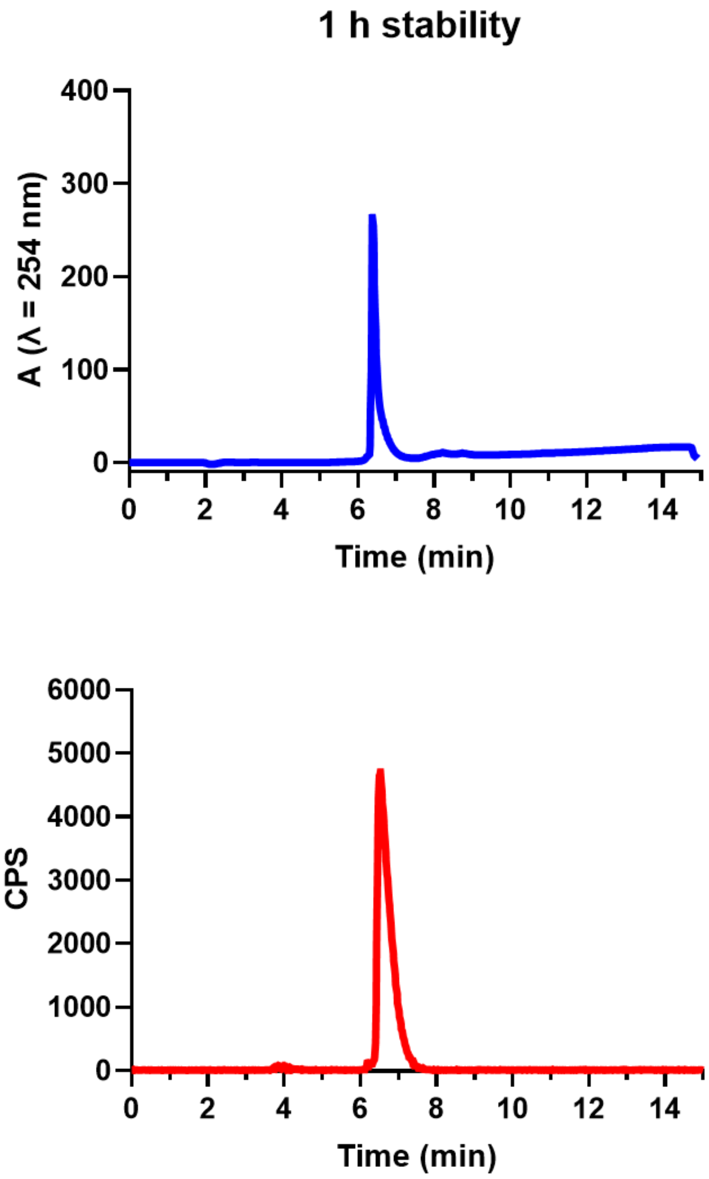

Figure S8. HPLC analysis of **Pt-succ-DFO-^{68}Ga** 1 h after radiolabeling showing stability of **Pt-succ-DFO-^{68}Ga** to photoreduction by radiolysis both in the UV-vis (top panel) and radioactivity (bottom panel) traces.

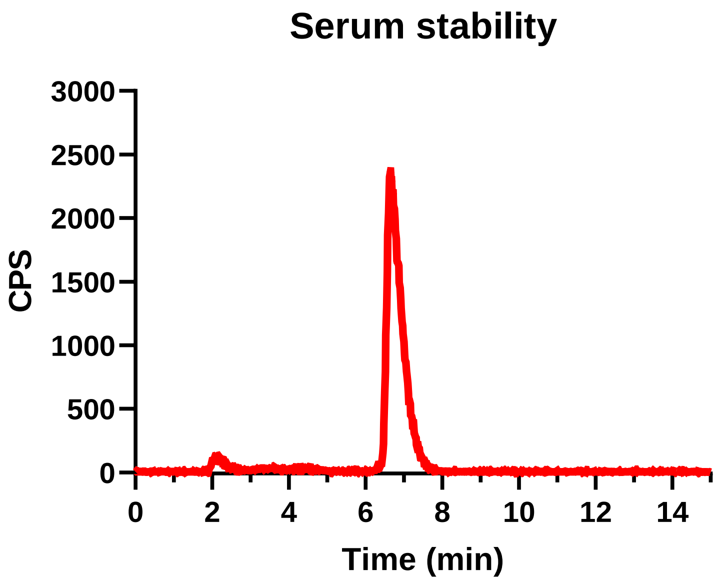

Figure S9. Radio-HPLC analysis of **Pt-succ-DFO-^{68}Ga** after 1 h incubation in human serum.

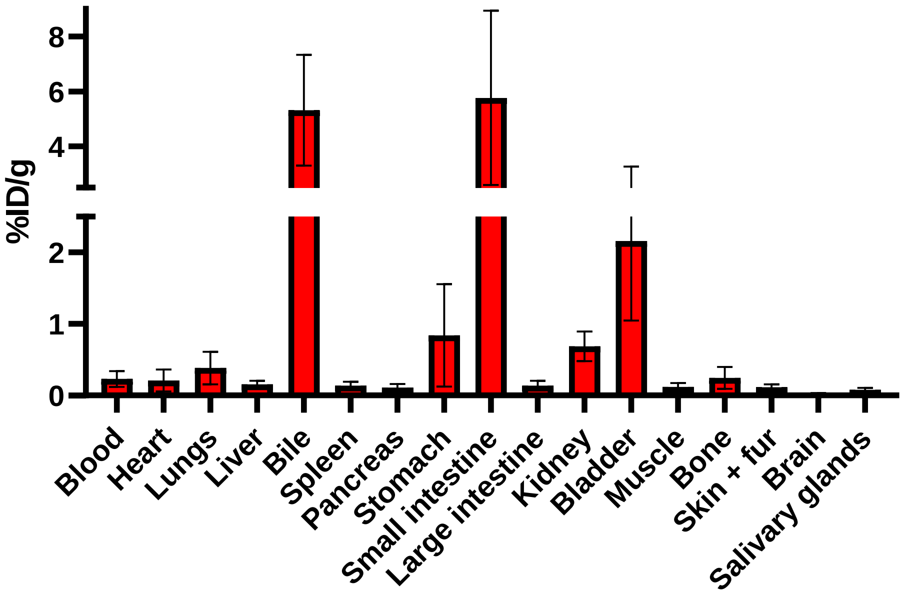

Figure S10. *Ex vivo* biodistribution of **Pt-succ-DFO-^{68}Ga** determined by γ-counting of selected organs at 2 h post injection (n = 4, mean ± SEM are reported).

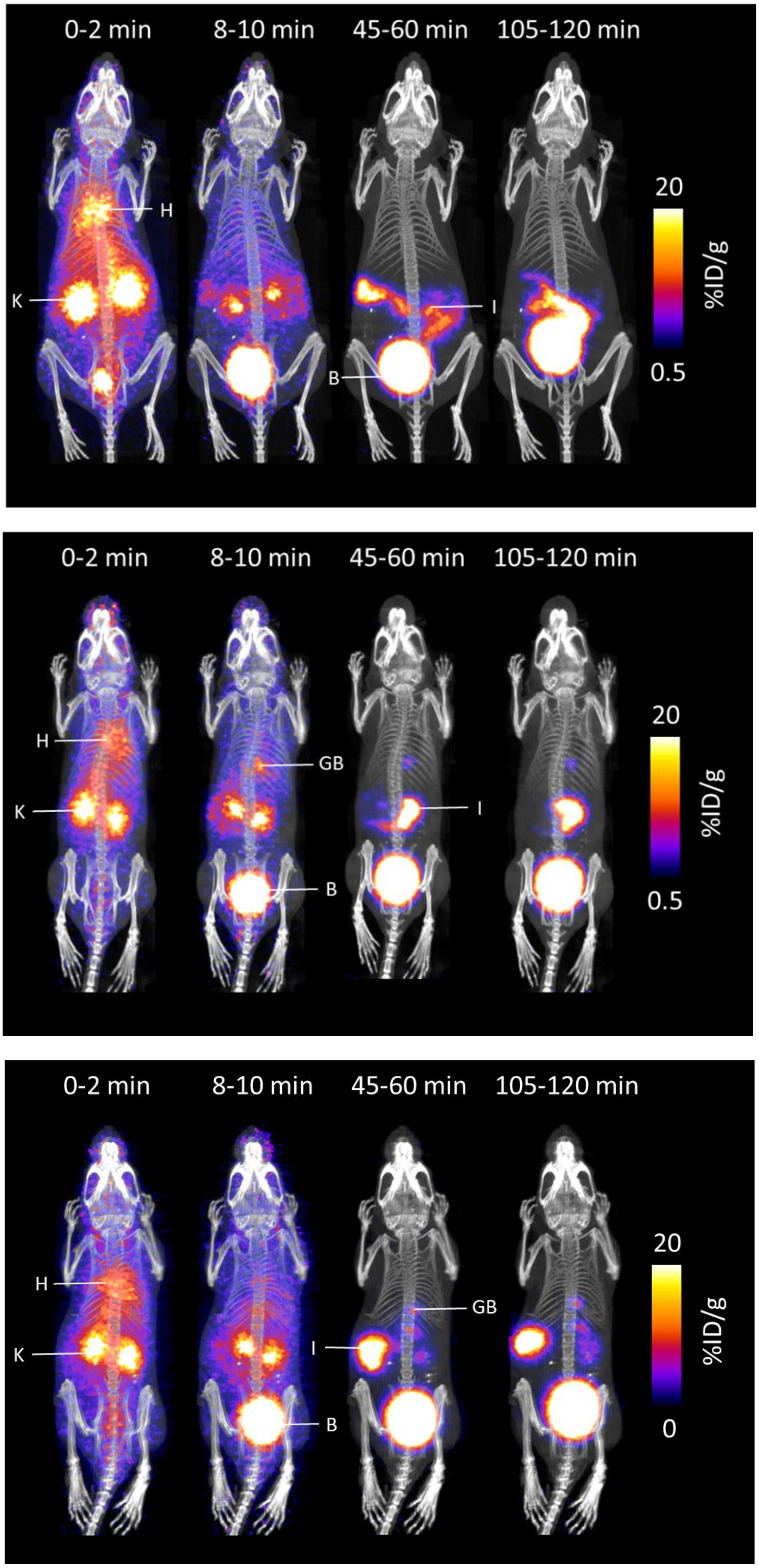

Figure S11. MIP images of **Pt-succ-DFO-^{68}Ga** biodistribution in three healthy animals at different time points after injection. H = heart, K = kidney, B = bladder, GB = gallbladder, I = intestine.
